# Frailty in community-dwelling older people: comparing screening instruments

**DOI:** 10.11606/s1518-8787.2020054002114

**Published:** 2020-11-17

**Authors:** Jair Almeida Carneiro, Andressa Samantha Oliveira Souza, Luciana Colares Maia, Fernanda Marques da Costa, Edgar Nunes de Moraes, Antônio Prates Caldeira

**Affiliations:** I Universidade Estadual de Montes Claros Centro de Ciências Biológicas e da Saúde Departamento de Saúde Mental e Saúde Coletiva Montes ClarosMG Brasil Universidade Estadual de Montes Claros. Centro de Ciências Biológicas e da Saúde. Departamento de Saúde Mental e Saúde Coletiva. Montes Claros, MG, Brasil; II Universidade Estadual de Montes Claros Centro de Ciências Biológicas e da Saúde Montes ClarosMG Brasil Universidade Estadual de Montes Claros. Centro de Ciências Biológicas e da Saúde. Montes Claros, MG, Brasil; III Universidade Estadual de Montes Claros Centro de Ciências Biológicas e da Saúde Departamento de Clínica Médica Montes ClarosMG Brasil Universidade Estadual de Montes Claros. Centro de Ciências Biológicas e da Saúde. Departamento de Clínica Médica. Montes Claros, MG, Brasil; IV Universidade Estadual de Montes Claros Centro de Ciências Biológicas e da Saúde Departamento de Enfermagem Montes ClarosMG Brasil Universidade Estadual de Montes Claros. Centro de Ciências Biológicas e da Saúde. Departamento de Enfermagem. Montes Claros, MG, Brasil; V Universidade Federal de Minas Gerais Faculdade de Medicina Departamento de Clínica Médica Belo HorizonteMG Brasil Universidade Federal de Minas Gerais. Faculdade de Medicina. Departamento de Clínica Médica. Belo Horizonte, MG, Brasil; VI Universidade Estadual de Montes Claros Centro de Ciências Biológicas e da Saúde Departamento de Saúde da Mulher e da Criança Montes ClarosMG Brasil Universidade Estadual de Montes Claros. Centro de Ciências Biológicas e da Saúde. Departamento de Saúde da Mulher e da Criança. Montes Claros, MG, Brasil

**Keywords:** Aged, Frailty, Epidemiology, Reproducibility of Results, Risk Factors, Health Surveys, Instrumentation

## Abstract

**OBJECTIVE:**

To compare the Edmonton Frail Scale (EFS) and Clinical-Functional Vulnerability Index-20 (CFVI-20) instruments regarding degree of agreement and correlation and compare descriptive models with frailty-associated variables in community-dwelling older people in Brazil.

**METHODS:**

Cross-sectional study, nested in a population-based and household cohort. Baseline sampling was calculated based on a probabilistic approach by conglomerate in two stages. In the first stage, census tract was used as sampling unit. In the second, the number of households was defined according to the population density of individuals aged ≥ 60 years. The Kappa statistic evaluated the agreement between instruments and Pearson's coefficient their correlation. Factors associated with frailty and high risk of clinical-functional vulnerability were identified by multiple analysis of Poisson regression with robust variance.

**RESULTS:**

Kappa statistics was 0.599 and Pearson's correlation coefficient 0.755 (p < 0.001). The EFS found a 28.2% prevalence of frailty, and the CFVI-20 found a 19.5% prevalence of high risk of clinical-functional vulnerability. Age equal to or greater than 80 years, history of stroke, polypharmacy, negative self-perceived health, fall in the past 12 months, and hospitalization in the past 12 months were variables associated with frailty in both instruments after multiple analysis. Less than four years of education, osteoarticular disease, and weight loss were associated with frailty only by EFS, and having a caregiver was associated with a high risk of clinical-functional vulnerability only by CFVI-20.

**CONCLUSIONS:**

Although the analyses show moderate agreement and strong positive correlation between the instruments, the indicated prevalence of frailty is discrepant. Our results attest the need to standardize the instrument for assessing frailty in community-dwelling older people.

## INTRODUCTION

By entailing a complex interaction of biological, psychological, and social factors, frailty in older people is a clinically recognizable multidimensional syndrome resulting from a decrease in energy reserves and age-related changes^1^^–^^3^. It often affects older adults with disproportionate health condition changes after stressful events, causing adverse clinical outcomes, such as impairment in activities of daily living, physical limitation, falls, hospitalization, and even death^2^^,^^4^^–^^6^.

The prevalence of frailty is expected to increase considerably with the population dynamics expected for the coming years^2^^,^^4^. Identifying frail older adults or those at-risk of frailty is a public health priority. Further appropriate interventions are required to reverse this condition severity or, for those whose condition is irreversible, reduce adverse outcomes^7^.

The Comprehensive Geriatric Assessment is the most appropriate strategy to identify and classify frail older adults^3^^,^^4^^,^^8^. It enables the identification of conditions that compromise patients’ health for developing a management plan addressing these conditions^4^^,^^9^. However, this specialized assessment method is considered complex and costly, especially when applied without distinction in community-dwelling older people^3^^,^^8^^,^^9^.

Although challenging, finding different ways of identifying frailty in community context is necessary due to the high cost incurred by older adults’ care in inappropriate places. Patients must be referred for the appropriate place for care, according to their needs. Several simple, fast-tracking instruments were developed^5^^,^^10^^,^^11^, but selecting from among them is difficult due to the lack of standard measure for frailty^5^. Besides that, the reliability and validity of most of them were not assessed^5^^,^^10^.

Among instruments following the best practices for complex measures development, we may stress the Edmonton Frail Scale (EFS)^10^ – an easy handling and simple application clinical proposal, even for professionals not specialized in geriatrics or gerontology^12^^,^^13^. Recently, the Clinical-Functional Vulnerability Index-20 (CFVI-20) was also developed in Brazil. Despite presenting a high degree of validity and reliability^14^, it is still little employed by researchers and health professionals.

EFS and CFVI-20 were not yet simultaneously employed in the same community-dwelling older population, and few studies compared these instruments with others serving the same purpose^15^^–^^20^. Comparing two tests allow us to investigate evidence of convergent validity; that is, the degree of agreement between the measured constructs. Given that both instruments assess the same construct and were validated by the Comprehensive Geriatric Assessment, we could expect a high degree of correlation. This study aims to compare EFS and CFVI-20 regarding the degree of agreement and correlation and compare descriptive models with frailty-associated variables in community-dwelling older people in Brazil.

## METHODS

This is a cross-sectional study nested with a population-based cohort and conducted with community-dwelling older people from the municipality of Montes Claros, in the north of Minas Gerais, Brazil. The municipality has approximately 400,000 inhabitants and is the main urban hub within the region.

Baseline sampling was calculated between May and July 2013 based on a probabilistic approach by conglomerate, in two stages. In the first stage, census tract was used as sampling unit. In the second, the number of households was defined according to the population density of individuals aged ≥ 60 years.

Our research data refer to the study first wave and were collected between November 2016 and February 2017. At this stage, the residence of all older adults interviewed at baseline was considered eligible for the new interview. As oriented by data collection instruments, older adults unable to answer the questionnaire were supported by family members or caregivers^12^^–^^14^.

EFS assesses nine domains (cognition, general health status, functional independence, social support, medication, nutrition, mood, urinary incontinence, and functional performance) distributed into 11 items with scores ranging from 0 to 17. Final score from 0 to 4 indicates no frailty; 5 and 6 indicate vulnerability to frailty; 7 and 8 mild frailty; 9 and 10, moderate frailty; and 11 or more indicate severe frailty^12^^,^^13^.

The CFVI-20 is a multidimensional assessment instrument containing 20 items that cover eight predictors of clinical-functional decline in older adults (age, self-perceived health, functional disabilities, cognition, mood, mobility, communication, and multiple comorbidities)^14^. Its score ranges from 0 to 40. Final score from 0 to 6 points indicates low risk of clinical-functional vulnerability; from 7 to 14 moderate risk; and 15 or higher indicate high risk, potentially frail^21^.

Dependent variables results were dichotomized at two levels: no frailty (final score ≤ 6) and frailty (final score > 6) according to the EFS; and no frailty (final score < 15) and frailty (final score ≥ 15) according to the CFVI-20. Independent variables were also dichotomized: gender, age group (up to 79 years or ≥ 80 years), marital status (with or without a partner), family arrangement (living alone or accompanied), education level (up to or more than four years of education), literacy (can read or not), own income (yes or no), household monthly income (up to or more than one minimum wage), self-reported chronic morbidities (hypertension, diabetes mellitus, heart disease, osteoarticular disease, neoplasia, stroke), polypharmacy (yes or no) and self-perceived health – assessed by the question “How would you rate your health status?”, with the following response options: “very good,” “good,” “fair,” “poor” or “very poor”.

Positive self-perceived health was classified as “very good” and “good” responses, while “fair,” “poor,” and “very poor” were classified as negative^22^^,^^23^. Self-reported weight loss in the past three months (yes or no), presence of caregiver (yes or no), fall in the past 12 months (yes or no), and hospitalization in the past 12 months (yes or no) were also evaluated.

Bivariate analyses were performed in both scales using the chi-square test to identify factors associated with response variable. Poisson regression with robust variance was used to calculate adjusted prevalence ratios (PR), considering independent variables associated with frailty in the bivariate analysis up to 20% significance level (p< 0.20). Analyses were performed separately for each instrument.

Considering frailty dichotomization (fragile × non-fragile), kappa statistics were applied to verify the agreement between EFS and CFVI-20 and interpreted according to Landis and Koch^24^. Instruments correlation was assessed based on the total scores, using Pearson's coefficient^25^. A significance level of 5% (p < 0.05) was set for all analyses. Collected data were analyzed using the Statistical Package for the Social Sciences (SPSS) version 17.0 (SPSS for Windows, Chicago, USA).

All participants were provided with information on the research and agreed to participate by signing an informed consent form. The project was approved by the Research Ethics Committee of the Faculdades Integradas Pitágoras de Montes Claros under the Opinion No. 1,629,395.

## RESULTS

Among the 685 older adults evaluated at baseline, 92 refused to participate in the second stage of the study, 78 changed residence and could not be located, 67 were not found at home after three visits, and 54 had died. Then, 394 community-dwelling older adults participated in the study. The most predominant age group was between 60 and 79 years, representing 76.6% of the sample, with mean age of 73.9 years (SD = 7.9).

In total, 66.8% were female, 50.6% lived alone, and 74.9% had up to four years of education; 88.3% did not have a caregiver, 71.3% had hypertension, and 48% had osteoarticular diseases. Table 1 shows sample characteristics and bivariate analyses results.

**Table 1:** Demographic, social, economic, and morbidity characterization, health-related care, and frailty-associated factors in community-dwellers older adults (bivariate analysis), 2017.

Independent variables		Sample		Frailty in the Edmonton Frail Scale (n = 394)					Frailty in the Clinical-Functional Vulnerability Index-20 (n = 394)				
		n	%	Yes		No		p	Yes		No		p
				n	%	n	%		n	%	n	%	
Gender								0.982					0.871
	Male	131	33.2	37	28.2	94	71.8		25	19.1	106	80.9	
	Female	263	66.8	74	28.1	189	71.9		52	19.8	211	80.2	
Age group								< 0.001					< 0.001
	Up to 79 years	302	76.6	70	23.2	232	77.8		35	11.6	267	88.4	
	≥ 80 years	92	23.4	41	44.6	51	55.4		42	45.7	50	54.3	
Marital status								0.378					0.039
	With partner	195	49.5	51	26.2	144	73.8		30	15.4	165	84.6	
	Without partner	199	50.6	60	30.2	139	69.8		47	23.6	152	76.4	
Family arrangement								0.977					0.218
	Living alone	50	12.7	14	28.0	36	72.0		13	26.0	37	74.0	
	Living accompanied	344	87.3	97	28.2	247	71.8		64	18.6	280	81.4	
Education level								< 0.001					0.002
	Up to 4 years	295	74.9	100	33.9	195	66.1		68	23.1	227	76.9	
	> 4 years	99	25.1	11	11.1	88	88.9		9	9.1	90	90.9	
Literacy								0.001					0.023
	Yes	300	76.1	72	24.0	228	76.0		51	17.0	249	83.0	
	No	94	23.9	39	41.5	55	58.5		26	27.7	68	72.3	
Own income								0.263					0.123
	No	39	9.9	8	20.5	31	79.5		4	10.3	35	89.7	
	Yes	355	90.1	103	29.0	252	71.0		73	20.6	282	79.4	
Household monthly income								0.109					0.040
	Up to one minimum wage	102	25.9	35	34.3	67	65.7		27	26.5	75	73.5	
	> one minimum wage	292	74.1	76	26.0	216	74.0		50	17.1	242	82.9	
Hypertension								0.029					0.047
	Yes	281	71.3	88	31.3	193	68.7		62	22.1	219	77.9	
	No	113	28.7	23	20.4	90	79.6		15	13.3	98	86.7	
Diabetes mellitus								0.215					0.631
	Yes	90	22.8	30	33.3	60	66.7		16	17.8	74	82.2	
	No	304	77.2	81	26.6	223	73.4		61	20.1	243	79.9	
Heart disease								0.003					< 0.001
	Yes	110	27.9	43	39.1	67	60.9		35	31.8	75	68.2	
	No	284	72.1	68	23.9	216	76.1		42	14.8	242	85.2	
Osteoarticular disease								0.002					0.040
	Yes	189	48.0	67	35.4	122	64.6		45	23.8	144	76.2	
	No	205	52.0	44	21.5	161	78.5		32	15.6	173	84.4	
Neoplasia								0.045					0.001
	Yes	38	9.6	16	42.1	22	57.9		15	39.5	23	60.5	
	No	356	90.4	95	26.7	261	73.3		62	17.4	294	82.6	
Cerebrovascular accident								0.001					< 0.001
	Yes	29	7.4	16	55.2	13	44.8		13	44.8	16	55.2	
	No	365	92.6	95	26.0	270	74.0		64	17.5	301	82.5	
Polypharmacy								< 0.001					< 0.001
	Yes	107	27.2	53	49.5	54	50.5		35	32.7	72	67.3	
	No	287	72.8	58	20.2	229	79.8		42	14.6	245	85.4	
Self-perceived health								< 0.001					< 0.001
	Negative	207	52.5	90	43.5	117	56.5		62	30.0	145	70.0	
	Positive	187	47.5	21	11.2	166	88.8		15	8.0	172	92.0	
Weight loss								< 0.001					0.001
	Yes	59	15.0	31	52.5	28	47.5		21	35.6	38	64.4	
	No	335	85.0	80	23.9	255	76.1		56	16.7	279	83.3	
Presence of caregiver								< 0.001					< 0.001
	Yes	46	11.7	25	54.3	21	45.7		23	50.0	23	50.0	
	No	348	88.3	86	24.7	262	75.3		54	15.5	294	84.5	
Fall in the past 12 months								< 0.001					< 0.001
	Yes	123	31.2	54	43.9	69	56.1		39	31.7	84	68.3	
	No	271	68.8	57	21.0	214	79.0		38	14.0	233	86.0	
Hospitalization in the past 12 months								< 0.001					< 0.001
	Yes	57	14.5	33	57.9	24	42.1		22	38.6	35	61.4	
	No	337	85.5	78	23.1	259	76.9		55	16.3	282	83.7	

The EFS found a 28.2% prevalence of frailty, and the CFVI-20 found a 19.5% prevalence of high risk of clinical-functional vulnerability (equivalent to frailty in EFS). Table 2 shows the frequency distribution of EFS components and Table 3 of CFVI-20 components.

**Table 2:** Frequency of Edmonton Frail Scale components in community-dwellers older adults, 2017.

Edmonton Frail Scale components		n	%
Cognition (clock drawing test)	Accepted	78	19.8
	Rejected with minor mistakes	64	16.2
	Rejected with major mistakes	252	64.0
General health status (hospitalization in the past 12 months)	None	337	85.5
	1 to 2	48	12.2
	More than 2	9	2.3
Self-perceived health	Excellent/very good/good	187	47.5
	Poor	180	45.7
	Very poor	27	6.8
Functional independence (activities in which assistance is required)	0–1	267	67.8
	2–4	123	31.2
	5–8	4	1.0
Social support (when assistance is needed, the older adult has someone to count on)	Always	332	84.3
	Sometimes	57	14.5
	Never	5	1.2
Medication (five or more)	No	287	72.8
	Yes	107	27.2
Forget to take a medication	No	269	68.3
	Yes	125	31.7
Nutrition (weight loss)	No	335	85.0
	Yes	59	15.0
Mood (feel sad or depressed)	No	297	75.4
	Yes	97	24.6
Urinary incontinence	No	298	75.6
	Yes	96	24.4
Functional performance (Timed Up & Go test)	0–10 seconds	121	30.7
	11–20 seconds	189	48.0
	More than 20 seconds	84	21.3

**Table 3:** Frequency of Clinical-Functional Vulnerability Index-20 components in community-dwellers older adults, 2017.

Clinical-Functional Vulnerability Index-20 components				n	%
AGE		60 to 74 years old		226	57.4
		75 to 84 years old		128	32.5
		≥ 85 years		40	10.1
SELF-PERCEIVED HEALTH	Health compared to other people from the same age group	Excellent/very good/good		226	57.4
		Fair or bad		168	42.6
ACTIVITIES OF DAILY LIVING (ADLs)	Instrumental (ADL)	Stopped grocery shopping	Yes	85	21.6
			No	309	78.4
		Stopped managing finances	Yes	71	18.1
			No	323	81.9
		Stopped performing minor housework	Yes	80	20.3
			No	314	79.7
	Basic (ADL)	Stopped bathing alone	Yes	24	6.1
			No	370	93.9
COGNITION	Forgetfulness		Yes	103	26.1
			No	291	73.9
	Worsening of forgetfulness in the past months		Yes	68	17.3
			No	326	82.7
	Forgetfulness preventing the performance of daily activities		Yes	55	14.0
			No	339	86.0
MOOD	Dismay, sadness, or hopelessness in the past month		Yes	109	27.7
			No	285	72.3
	Loss of interest or pleasure in the past month in previously enjoyable activities		Yes	81	20.6
			No	313	79.4
MOBILITY	Reach, graspingness, and pincer grip	Inability to raise the arm above shoulder level	Yes	35	8.9
			No	359	91.1
		Inability to handle or hold small objects	Yes	31	7.8
			No	363	92.2
	Aerobic and muscle capacity	Unintentional weight loss, BMI < 22 kg/m2, calf circumference < 31 cm, or gait speed (4 m) > 5 sec.	Yes	49	12.4
			No	345	87.6
	Gait	Walking difficulties preventing to perform some daily activities	Yes	109	27.7
			No	285	72.3
		Two or more falls in the past year	Yes	110	27.9
			No	284	72.1
	Sphincteral incontinence	Involuntary loss of urine or feces	Yes	117	29.7
			No	277	70.3
COMMUNICATION	Vision	Vision impairment that may prevent the performance of some daily activities	Yes	80	20.3
			No	314	79.7
	Hearing	Hearing impairment that may prevent the performance of some daily activities	Yes	79	20.1
			No	315	79.9
MULTIPLE COMORBIDITIES	Polypathology	≥ 5 chronic diseases ≥ 5 daily medications Hospitalization in the past 6 months	Yes	83	21.1
	Polypharmacy				

In EFS, 190 older adults (48.2%) presented no frailty, 93 (23.6%) were apparently vulnerable to frailty, 74 (18.8%) had mild frailty, 32 (8.1%) moderate frailty, and 5 (1.3%) severe fragility. As for the CFVI-20, 207 (52.5%) were robust, or with low risk of frailty, 110 (28.0%) had moderate risk of clinical-functional vulnerability, and 77 (19.5%) high risk.

Kappa statistics found a 0.599 agreement index between the instruments (Table 4). Pearson's correlation coefficient between EFS and CFVI-20 was 0.755 (p < 0.001).

**Table 4:** Analysis of agreement for frailty classification according to Edmonton Frail Scale and Clinical-Functional Vulnerability Index-20 in community-dwellers older adults, 2017.

	Edmonton Frail Scale (EFS)				Total
	No frailty		Frailty		
CFVI-20:	(n)	(%)	(n)	(%)	
No frailty	271	85.5	46	14.5	317
Frailty	12	15.6	65	84.4	77

Kappa = 0.599 (p < 0.001).

Age equal to or greater than 80 years, history of stroke, polypharmacy, negative self-perceived health, fall in the past 12 months, and hospitalization in the past 12 months were variables that remained statistically associated with frailty in both instruments after multiple analysis. Less than four years of education, osteoarticular disease, and weight loss were associated with frailty only by EFS, and having a caregiver was associated with a higher risk of fragility only by CFVI-20 (Table 5).

**Table 5:** Frailty-associated factors in community-dwellers older adults according to Edmonton Frail Scale and Clinical-Functional Vulnerability Index-20 (multiple analysis), 2017.

Independent variables		Frailty in the Edmonton Frail Scale			Frailty in the CFVI-20		
		PR	95%CI	p	PR	95%CI	p
Age group				0.001			< 0.001
	≥ 80 years	1.643	1.239 – 2.178		3.327	2.204 – 5.021	
	Up to 79 years	1			1		
Education level				0.002			
	Up to 4 years	2.171	1.314 – 3.589				
	> 4 years	1					
Osteoarticular disease				0.016			
	Yes	1.410	1.065 – 1.865				
	No	1					
Cerebrovascular accident				< 0.001			< 0.001
	Yes	2.139	1.484 – 3.082		2.546	1.619 – 4.004	
	No	1			1		
Polypharmacy				0.001			0.004
	Yes	1.610	1.217 – 2.130		1.657	1.174 – 2.337	
	No	1			1		
Self-perceived health				< 0.001			< 0.001
	Negative	3.115	2.085 – 4.654		3.294	2.081 – 5.213	
	Positive	1			1		
Weight loss				0.006			
	Yes	1.542	1.132 – 2.102				
	No	1					
Presence of caregiver							0.020
	Yes				1.615	1.078 – 2.419	
	No				1		
Fall in the past 12 months				0.037			0.029
	Yes	1.363	1.019 – 1.824		1.503	1.043 – 2.166	
	No	1			1		
Hospitalization in the past 12 months				< 0.001			0.005
	Yes	1.825	1.382 – 2.409		1.715	1.181 – 2.490	
	No	1			1		

PR: prevalence ratio.

## DISCUSSION

We found a moderate agreement and a strong positive correlation between EFS and CFVI-20. The prevalence of frailty in community-dwelling older people was higher in EFS. Demographic, social, economic, and morbidity-related factors, as well as health services use, influenced frailty in community-dwelling older people, but differences within the identification of these variables by the instruments was small.

The similarity and relevance of the main components justify the moderate agreement found between the instruments. Both scales assess cognition, functional independence, mood, and health conditions (or presence of morbidities). The EFS separately assesses social support, medication, nutrition, urinary incontinence, and functional performance; in turn, CFVI-20 assesses age, self-perceived health, mobility, and communication^12^^–^^14^.

Our results differ from those reported by a systematic review and the meta-analysis of studies conducted in Latin America and the Caribbean^26^, where the prevalence of frailty identified by the EFS in Brazilian community-dwelling older adults was 35.8%, with 95%CI 30.6–41,2^26^. As for the CFVI-20, although validated in Brazil, few population-based studies employed it^14^.

The different prevalence found in both instruments may be explained by the cutoff point. ICVF-20 cut-off point refer fewer older adults for specialized evaluation by screening, identifying those with greater needs. Considering the benefit-cost ratio, this process is considered positive due to the high cost of broad geriatric assessment. Given that specialized care services are not always available, this is an opportunity to optimize resources in primary care.

Another possible explanation for the discrepancy between scales prevalence is the differences among some of their components: while EFS assesses “social support,” CFVI-20 approaches “age” and “communication.” Besides that, similar components are approached differently by each instrument. While the EFS assesses “cognition” using the clock drawing test, the CFVI-20 does so by evoking words. As the clock drawing test requires number knowledge, the low education level among Brazilians older adults may compromise its result. Thus, the low performance in this test (which increases the prevalence of frailty) may be related to difficulties not necessarily associated to a cognitive deficit^13^.

EFS assesses “health status” by the number of hospitalizations in the past 12 months; in turn, ICVF-20 addresses the number of hospitalizations in the past six months in the component “multiple comorbidities.” The instruments also differ regarding “functional independence,” or “functional disability”; while EFS approach it by preparing meals/cooking, getting around from place to place, using the phone, doing laundry, and taking medicines, CFVI-20 employs doing the dishes and bathing.

In the component “medication,” EFS approaches forgetting to take medications, which is unregarded by the CFVI-20. In EFS, “functional performance” is evaluated using the timed Up & Go Test with a distance of approximately three meters and time stratified by “0 to 10 seconds,” “11 to 20 seconds,” and “greater than 20 seconds.” CFVI-20, in turn, assesses whether the time spent on the 4-meter gait speed test is greater than five seconds.

CFVI-20 also differs from EFS by including the “mobility” component – which assesses the ability to raise the arms above the shoulder level and handle or hold small objects, Body Mass Index, calf circumference, walking difficulties that may interfere with activities of daily living, falls in the past year, and fecal incontinence – and addressing polypathology in the “multiple comorbidities” component.

These factors reveal that the instruments diverse characteristics influence the prevalence of frailty in older adults. A systematic review^27^ concluded that frailty components and corresponding indicators considerably vary depending on the method employed by the instrument. It also reported a lack of consensus regarding which elements should be considered to predict frailty and, consequently, increase this condition accurate diagnosis^27^.

Our results found a correlation between advanced age and frailty regardless of the instrument used. However, frailty correlation with low education was only identified by the EFS. Other studies comparing instruments^15^^,^^18^ also observed this association between frailty, advanced age, and lower education level. A longitudinal study conducted in the Netherlands identified, besides the association with low education, an association between low income and frailty^28^.

The history of stroke and falls – factors associated with frailty in both instruments, – as well as the osteoarticular disease identified by the EFS corroborate results reported by other studies^4^^,^^6^^,^^7^^,^^15^. Osteoarticular disease and stroke sequelae engender functional limitations that impair the performance of basic, instrumental, and advanced activities that were previously performed without restrictions, increasing the risk of falls.

We also found an association between polypharmacy and frailty in both instruments, a result confirmed in this condition consensus^3^ and also reported by other authors^15^^,^^28^^,^^29^. A French study found independent and combined effects of polypharmacy and frailty on mortality risk factors in older adults^28^. This vulnerability may be explained by drugs pharmacokinetic and pharmacodynamic properties in the aging body, as well as by the potential adverse reactions of drug interaction.

The two instruments also showed an association between frailty and negative self-perceived health – an indicator that incorporates physical, cognitive, and emotional components, as well as aspects related to well-being and personal life satisfaction^22^^,^^23^^,^^30^. Considering that, this measure can predict mortality, functional capacity decline, and frailty in older adults.

We also found an association between frailty and weight loss in the EFS. Impaired nutritional status is an important sign of frailty in older adults, and dietary intervention is a non-pharmacological treatment capable of correcting macro and micronutrient deficiency, preventing weight loss that can lead to frailty syndrome^7^.

Frailty and the presence of a caregiver were only associated in the CFVI-20 and probably indicates a reverse causality, that is: the frail older adult needs a caregiver to assist him in the activities of daily living^7^^,^^9^^,^^19^. Thus, caregivers demand or presence would be markers of existing fragility.

Hospitalization was associated with frailty in both instruments – a result also confirmed in meta-analysis^6^. Although chronic diseases are not necessarily accompanied by frailty, acute episodes of certain illnesses or exacerbation of chronic conditions may increase the risk of adverse events^7^, leading to frailty in older people and, consequently, to unfavorable clinical outcomes, such as hospitalization^2^^,^^6^. Hospitalizations for any reason cause important changes in older adults’ daily life.

Comparing instruments capable of identifying frailty in community-dwellers older adults may contribute to the search for an applicable instrument, especially at primary healthcare and places with few professionals specialized in geriatrics. Despite their peculiarities, both scales were akin in identifying associated factors or fragility markers and may be useful to health teams in outlining components that most interfere with fragility and in identifying older adults who require specialized care. The CFVI-20 seems more useful in a context of few resources, for determining a smaller number of patients to be referred for comprehensive geriatric assessment.

Our study has some limitations. The main limitation is the lack of a comprehensive geriatric assessment, which would allow other simultaneous analyses of the two instruments. However, this procedure was separately performed in the instruments validation. As this is a cross-sectional study, we could not establish causal relationships. Moreover, both instruments include self-reported components, relying on the memory of the interviewee or their caregiver. However, our study carefully evaluated a representative random sample of community-dwellers older adults using validated and reliable instruments.

## CONCLUSIONS

The EFS and CFVI-20 instruments showed moderate agreement and strong positive correlation, as well as similar features for identifying associations. However, the prevalence of frailty differed between them. This result stresses the need to standardize the instrument for measuring frailty in community-dwellers older adults.
