# Supplementary material for: Frailty in community-dwelling older people: comparing screening instruments
Source: Rev Saude Publica. 2020 Nov 17;54:119. doi: 10.11606/s1518-8787.2020054002114 (PMC7671582; doi:10.11606/s1518-8787.2020054002114)
Supplement: Translation

# Translation: Fragilidade em idosos comunitários: comparando instrumentos de triagem

**Authors:** Jair Almeida Carneiro, Andressa Samantha Oliveira Souza, Luciana Colares Maia, Fernanda Marques da Costa, Edgar Nunes de Moraes, Antônio Prates Caldeira

**Affiliations:** I Montes ClarosMG Brasil Universidade Estadual de Montes Claros. Centro de Ciências Biológicas e da Saúde. Departamento de Saúde Mental e Saúde Coletiva. Montes Claros, MG, Brasil; II Montes ClarosMG Brasil Universidade Estadual de Montes Claros. Centro de Ciências Biológicas e da Saúde. Montes Claros, MG, Brasil; III Montes ClarosMG Brasil Universidade Estadual de Montes Claros. Centro de Ciências Biológicas e da Saúde. Departamento de Clínica Médica. Montes Claros, MG, Brasil; IV Montes ClarosMG Brasil Universidade Estadual de Montes Claros. Centro de Ciências Biológicas e da Saúde. Departamento de Enfermagem. Montes Claros, MG, Brasil; V Belo HorizonteMG Brasil Universidade Federal de Minas Gerais. Faculdade de Medicina. Departamento de Clínica Médica. Belo Horizonte, MG, Brasil; VI Montes ClarosMG Brasil Universidade Estadual de Montes Claros. Centro de Ciências Biológicas e da Saúde. Departamento de Saúde da Mulher e da Criança. Montes Claros, MG, Brasil

**Keywords:** Idoso, Fragilidade, Epidemiologia, Reprodutibilidade dos Testes, Fatores de Risco, Inquéritos Epidemiológicos, Instrumentação

## Abstract

**OBJETIVO:**

Comparar os instrumentos Edmonton Frail Scale (EFS) e Índice de Vulnerabilidade Clínico-Funcional-20 (IVCF-20) quanto ao grau de concordância e de correlação, bem como comparar modelos descritivos com variáveis associadas à fragilidade em idosos brasileiros comunitários.

**MÉTODOS:**

Estudo transversal, aninhado a uma coorte de base populacional e domiciliar. A amostragem na linha de base foi probabilística, por conglomerados, em dois estágios. No primeiro, utilizou-se como unidade amostral o setor censitário. No segundo, definiu-se o número de domicílios segundo a densidade populacional de indivíduos com idade ≥ a 60 anos. A estatística Kappa analisou a concordância, e o coeficiente de Pearson avaliou a correlação entre os instrumentos. Os fatores associados à fragilidade e ao alto risco de vulnerabilidade clínico-funcional foram identificados por análise múltipla de regressão de Poisson com variância robusta.

**RESULTADOS:**

A estatística Kappa foi 0,599, e o coeficiente de correlação de Pearson foi de 0,755 (p < 0,001). A prevalência da fragilidade foi de 28,2% pela EFS, e a prevalência do alto risco de vulnerabilidade clínico-funcional foi de 19,5% pelo IVCF-20. As variáveis associadas à fragilidade, após análise múltipla, em ambos os instrumentos, foram: idade igual ou superior a 80 anos, histórico de acidente vascular encefálico, polifarmácia, autopercepção negativa de saúde, queda nos últimos 12 meses e internação nos últimos 12 meses. Escolaridade inferior a quatro anos, doença osteoarticular e perda de peso foram associadas a fragilidade apenas pela EFS, enquanto possuir cuidador esteve associado a alto risco de vulnerabilidade clínico-funcional somente pelo IVCF-20.

**CONCLUSÕES:**

Embora as análises revelem concordância moderada e forte correlação positiva entre os instrumentos, a prevalência de fragilidade apontada é discrepante. O resultado destaca a necessidade de padronizar o instrumento para aferir a fragilidade em idosos comunitários.

## INTRODUÇÃO

Envolvendo uma interação complexa de fatores biológicos, psicológicos e sociais, a fragilidade em idosos é uma síndrome multidimensional, clinicamente reconhecível, que resulta da diminuição de reservas energéticas e de alterações relacionadas ao envelhecimento^1^^–^^3^. Ela é geralmente observada em idosos que apresentam mudanças desproporcionais no estado de saúde após eventos estressores, levando à ocorrência de desfechos clínicos adversos, como comprometimento das atividades da vida diária, limitação física, quedas, hospitalização e até óbito^2^^,^^4^^–^^6^.

A prevalência da fragilidade deve aumentar consideravelmente com a dinâmica populacional esperada para os próximos anos^2^^,^^4^. Identificar idosos frágeis ou em risco de fragilização é prioridade em saúde pública. Intervenções apropriadas subsequentes são necessárias para reverter a gravidade do quadro ou, em caso de pacientes cuja condição é irreversível, reduzir desfechos adversos^7^.

A Avaliação Geriátrica Abrangente é a mais adequada estratégia para detectar e classificar o idoso frágil^3^^,^^4^^,^^8^. Por meio dela, consegue-se identificar condições que comprometem a saúde do paciente e criar um plano de gestão para abordá-las^4^^,^^9^. Todavia, essa avaliação especializada é considerada complexa e dispendiosa, especialmente quando aplicada sem distinção em idosos comunitários^3^^,^^8^^,^^9^.

O desafio de encontrar outras formas de identificar a fragilidade no contexto comunitário precisa ser enfrentado, pois o custo da atenção ao idoso assistido em local inadequado é muito elevado. É fundamental encaminhar o paciente ao local adequado de assistência, conforme a necessidade apresentada. Vários instrumentos simples, de rastreio rápido, foram desenvolvidos^5^^,^^10^^,^^11^, e é difícil escolher dentre eles, uma vez que não há medida-padrão para a fragilidade^5^. Além disso, a confiabilidade e a validade da maioria desses instrumentos não foram avaliadas^5^^,^^10^.

Dentre os instrumentos que seguem as melhores práticas no desenvolvimento de medidas complexas, está a *Edmonton Frail Scale* (EFS)^10^, proposta clínica de fácil manuseio e aplicação, inclusive por profissionais não especializados em geriatria ou gerontologia^12^^,^^13^. Recentemente, no Brasil, foi desenvolvido também o Índice de Vulnerabilidade Clínico-Funcional-20 (IVCF-20), com alto grau de validade e confiabilidade^14^, mas ainda pouco utilizado por pesquisadores e profissionais da saúde.

A EFS e o IVCF-20 ainda não foram empregados simultaneamente em uma mesma população de idosos comunitários, e foram poucos os estudos que compararam esses instrumentos com outros de mesma finalidade^15^^–^^20^. A comparação entre dois testes permite investigar evidências de validade convergente, isto é, o grau de concordância entre os construtos medidos. Assumindo o pressuposto de que ambos os instrumentos avaliam o mesmo construto e foram igualmente validados a partir da Avaliação Geriátrica Abrangente, espera-se elevado grau de correlação. O presente estudo tem por objetivo comparar os instrumentos EFS e IVCF-20 quanto ao grau de concordância e de correlação, bem como comparar modelos descritivos com variáveis associadas à fragilidade em idosos brasileiros comunitários.

## MÉTODOS

Trata-se de estudo transversal aninhado a uma coorte de base populacional, realizado com idosos comunitários. A pesquisa foi desenvolvida no município de Montes Claros, norte de Minas Gerais, Brasil. A cidade conta com uma população de aproximadamente 400 mil habitantes e é o principal polo urbano de sua região.

O processo de amostragem na linha de base do estudo, ocorrido entre maio e julho de 2013, foi probabilístico, por conglomerados e em dois estágios. No primeiro estágio, utilizou-se como unidade amostral o setor censitário. No segundo, definiu-se o número de domicílios segundo a densidade populacional de indivíduos com idade maior ou igual a 60 anos.

Os dados da pesquisa referem-se à primeira onda do estudo e foram coletados entre os meses de novembro de 2016 e fevereiro de 2017. Nessa etapa, a residência de todos os idosos entrevistados na linha de base foi considerada elegível para a nova entrevista. Conforme orientação dos instrumentos de coleta de dados, as perguntas do questionário foram respondidas com o auxílio de familiares ou acompanhantes para os idosos incapazes de responder^12^^–^^14^.

A EFS avalia nove domínios (cognição, estado de saúde, independência funcional, suporte social, uso de medicamento, nutrição, humor, continência urinária e desempenho funcional), distribuídos em 11 itens com pontuação que varia de 0 a 17. O escore final de 0 a 4 indica que não há presença de fragilidade; 5 e 6 definem o idoso como aparentemente vulnerável para fragilidade; 7 e 8, com fragilidade leve; 9 e 10, com fragilidade moderada; e 11 ou mais pontos, fragilidade severa^12^^,^^13^.

O IVCF-20 é um instrumento multidimensional com 20 itens de avaliação que abrangem oito condições preditoras de declínio clínico-funcional do idoso (idade, autopercepção da saúde, incapacidades funcionais, cognição, humor, mobilidade, comunicação e comorbidades múltiplas)^14^. A pontuação varia de 0 a 40. O escore final de 0 a 6 pontos indica idoso com baixo risco de vulnerabilidade clínico-funcional; de 7 a 14, moderado risco; e 15 ou mais, alto risco, potencialmente frágil^21^.

Os resultados das variáveis dependentes foram dicotomizados em dois níveis: sem fragilidade (escore final ≤ 6) e com fragilidade (escore final > 6), segundo a EFS, e sem fragilidade (escore final < 15) e com fragilidade (escore final ≥ 15) segundo a avaliação do IVCF-20. As variáveis independentes estudadas foram igualmente dicotomizadas: sexo, faixa etária (até 79 anos ou ≥ 80 anos), situação conjugal (com ou sem companheiro), arranjo familiar (residir sozinho ou acompanhado), escolaridade (até quatro anos de estudo ou mais que quatro anos de estudo), alfabetização (sabe ler ou não), renda própria (sim ou não), renda familiar mensal (até um salário mínimo ou mais que um salário mínimo), presença ou ausência de morbidades crônicas autorreferidas (hipertensão arterial, diabetes mellitus, doença cardíaca, doença osteoarticular, neoplasia, acidente vascular encefálico), polifarmácia (sim ou não) e autopercepção de saúde, avaliada por meio da questão “Como o(a) sr.(a). classificaria seu estado de saúde?”, cujas respostas possíveis eram “muito bom”, “bom”, “regular”, “ruim” ou “muito ruim”.

Assumiu-se como percepção positiva da saúde as respostas “muito bom” e “bom”, enquanto as respostas “regular”, “ruim” e “muito ruim” foram classificadas como percepção negativa da saúde^22^^,^^23^. Avaliaram-se ainda: perda de peso autorreferida nos últimos três meses (sim ou não), presença de cuidador (sim ou não), queda nos últimos 12 meses (sim ou não) e internação nos últimos 12 meses (sim ou não).

Em ambas as escalas, análises bivariadas foram realizadas para identificar fatores associados à variável-resposta, por meio do teste qui-quadrado. Utilizou-se a regressão de Poisson, com variância robusta, para calcular as razões de prevalência (RP) ajustadas, considerando as variáveis independentes associadas à fragilidade na análise bivariada, até o nível de significância de 20% (p < 0,20). As análises foram realizadas separadamente para cada instrumento.

Para verificar a concordância entre os instrumentos EFS e IVCF-20, considerando a dicotomização da fragilidade (frágil × não frágil), foi aplicada a estatística Kappa, interpretada conforme Landis e Koch^24^. Avaliou-se a correlação entre os instrumentos por meio do coeficiente de Pearson^25^, considerando os escores totais. Para todas as análises, definiu-se um nível de significância final de 5% (p < 0,05). As informações foram analisadas por meio do programa Statistical Package for the Social Sciences (SPSS), versão 17.0 (SPSS for Windows, Chicago, EUA).

Os participantes foram orientados sobre o estudo e apresentaram sua anuência com a assinatura de Termo de Consentimento Livre e Esclarecido. O Comitê de Ética em Pesquisa das Faculdades Integradas Pitágoras de Montes Claros aprovou o projeto por meio do Parecer Consubstanciado n° 1.629.395.

## RESULTADOS

Dentre os 685 idosos avaliados no ano-base, 92 se recusaram a participar da segunda fase do estudo, 78 mudaram de residência e não foram localizados, 67 não foram encontrados no domicílio após três visitas e 54 haviam falecido. Logo, participaram da pesquisa 394 idosos comunitários. Predominou a faixa etária entre 60 e 79 anos, representando 76,6% da amostra, com média de 73,9 anos (DP = 7,9).

Dos idosos, 66,8% eram do sexo feminino, 50,6% residiam sem companheiro, e 74,9% tinham até quatro anos de estudo; 88,3% não tinham um cuidador, 71,3% eram hipertensos, e 48% relataram doenças osteoarticulares. As características do grupo avaliado são apresentadas na Tabela 1, que mostra também os resultados das análises bivariadas.

**Tabela 1:** Caracterização demográfica, social, econômica e de morbidade, cuidados relacionados à saúde e fatores associados à fragilidade de idosos comunitários (análise bivariada), 2017.

Variáveis independentes		Amostra		Fragilidade *Edmonton Frail Scale* (n = 394)					Fragilidade Índice de Vulnerabilidade Clínico-Funcional-20 (n = 394)				
		n	%	Sim		Não		p	Sim		Não		p
				n	%	n	%		n	%	n	%	
Sexo								0,982					0,871
	Masculino	131	33,2	37	28,2	94	71,8		25	19,1	106	80,9	
	Feminino	263	66,8	74	28,1	189	71,9		52	19,8	211	80,2	
Faixa etária								< 0,001					< 0,001
	Até 79 anos	302	76,6	70	23,2	232	77,8		35	11,6	267	88,4	
	≥ 80 anos	92	23,4	41	44,6	51	55,4		42	45,7	50	54,3	
Situação conjugal								0,378					0,039
	Com companheiro	195	49,5	51	26,2	144	73,8		30	15,4	165	84,6	
	Sem companheiro	199	50,6	60	30,2	139	69,8		47	23,6	152	76,4	
Arranjo familiar								0,977					0,218
	Reside sozinho	50	12,7	14	28,0	36	72,0		13	26,0	37	74,0	
	Corresidente	344	87,3	97	28,2	247	71,8		64	18,6	280	81,4	
Escolaridade								< 0,001					0,002
	Até 4 anos	295	74,9	100	33,9	195	66,1		68	23,1	227	76,9	
	> 4 anos	99	25,1	11	11,1	88	88,9		9	9,1	90	90,9	
Sabe ler								0,001					0,023
	Sim	300	76,1	72	24,0	228	76,0		51	17,0	249	83,0	
	Não	94	23,9	39	41,5	55	58,5		26	27,7	68	72,3	
Renda própria								0,263					0,123
	Não	39	9,9	8	20,5	31	79,5		4	10,3	35	89,7	
	Sim	355	90,1	103	29,0	252	71,0		73	20,6	282	79,4	
Renda familiar mensal								0,109					0,040
	Até 1 salário	102	25,9	35	34,3	67	65,7		27	26,5	75	73,5	
	> de 1 salário	292	74,1	76	26,0	216	74,0		50	17,1	242	82,9	
Hipertensão arterial								0,029					0,047
	Sim	281	71,3	88	31,3	193	68,7		62	22,1	219	77,9	
	Não	113	28,7	23	20,4	90	79,6		15	13,3	98	86,7	
Diabetes mellitus								0,215					0,631
	Sim	90	22,8	30	33,3	60	66,7		16	17,8	74	82,2	
	Não	304	77,2	81	26,6	223	73,4		61	20,1	243	79,9	
Doença cardíaca								0,003					< 0,001
	Sim	110	27,9	43	39,1	67	60,9		35	31,8	75	68,2	
	Não	284	72,1	68	23,9	216	76,1		42	14,8	242	85,2	
Doença osteoarticular								0,002					0,040
	Sim	189	48,0	67	35,4	122	64,6		45	23,8	144	76,2	
	Não	205	52,0	44	21,5	161	78,5		32	15,6	173	84,4	
Neoplasia								0,045					0,001
	Sim	38	9,6	16	42,1	22	57,9		15	39,5	23	60,5	
	Não	356	90,4	95	26,7	261	73,3		62	17,4	294	82,6	
Acidente vascular encefálico								0,001					< 0,001
	Sim	29	7,4	16	55,2	13	44,8		13	44,8	16	55,2	
	Não	365	92,6	95	26,0	270	74,0		64	17,5	301	82,5	
Polifarmácia								< 0,001					< 0,001
	Sim	107	27,2	53	49,5	54	50,5		35	32,7	72	67,3	
	Não	287	72,8	58	20,2	229	79,8		42	14,6	245	85,4	
Autopercepção de saúde								< 0,001					< 0,001
	Negativa	207	52,5	90	43,5	117	56,5		62	30,0	145	70,0	
	Positiva	187	47,5	21	11,2	166	88,8		15	8,0	172	92,0	
Perda de peso								< 0,001					0,001
	Sim	59	15,0	31	52,5	28	47,5		21	35,6	38	64,4	
	Não	335	85,0	80	23,9	255	76,1		56	16,7	279	83,3	
Possui cuidador								< 0,001					< 0,001
	Sim	46	11,7	25	54,3	21	45,7		23	50,0	23	50,0	
	Não	348	88,3	86	24,7	262	75,3		54	15,5	294	84,5	
Queda nos últimos12 meses								< 0,001					< 0,001
	Sim	123	31,2	54	43,9	69	56,1		39	31,7	84	68,3	
	Não	271	68,8	57	21,0	214	79,0		38	14,0	233	86,0	
Internação nos últimos12 meses								< 0,001					< 0,001
	Sim	57	14,5	33	57,9	24	42,1		22	38,6	35	61,4	
	Não	337	85,5	78	23,1	259	76,9		55	16,3	282	83,7	

A prevalência da fragilidade foi de 28,2% pela EFS, e a prevalência do alto risco de vulnerabilidade clínico-funcional pelo IVCF-20 (equivalente à fragilidade no EFS) foi de 19,5%. A Tabela 2 mostra a distribuição de frequência dos componentes da EFS, e a Tabela 3 do IVCF-20.

**Tabela 2:** Frequência dos componentes da *Edmonton Frail Scale* em idosos comunitários, 2017.

Componentes da *Edmonton Frail Scale*		n	%
Cognição (teste do desenho do relógio)	Aprovado	78	19,8
	Reprovado com erros mínimos	64	16,2
	Reprovado com erros significativos	252	64,0
Estado geral de saúde (internação nos últimos 12 meses)	Nenhuma	337	85,5
	1 a 2	48	12,2
	Maior que 2	9	2,3
Autopercepção de saúde	Excelente/Muito boa /Boa	187	47,5
	Razoável	180	45,7
	Ruim	27	6,8
Independência funcional (atividades em que precisa de ajuda)	0–1	267	67,8
	2–4	123	31,2
	5–8	4	1,0
Suporte social (quando precisa de ajuda, pode contar com alguém)	Sempre	332	84,3
	Algumas vezes	57	14,5
	Nunca	5	1,2
Uso de medicamentos (cinco ou mais)	Não	287	72,8
	Sim	107	27,2
Esquece de tomar medicamento	Não	269	68,3
	Sim	125	31,7
Nutrição (perda de peso)	Não	335	85,0
	Sim	59	15,0
Humor (triste ou deprimido)	Não	297	75,4
	Sim	97	24,6
Incontinência urinária	Não	298	75,6
	Sim	96	24,4
Desempenho funcional (“Levante e Ande” cronometrado)	0–10 segundos	121	30,7
	11–20 segundos	189	48,0
	Maior que 20 segundos	84	21,3

**Tabela 3:** Frequência dos componentes do Índice de Vulnerabilidade Clínico-Funcional-20 em idosos comunitários, 2017.

Componentes do Índice de Vulnerabilidade Clínico-Funcional-20				n	%
IDADE		60 a 74 anos		226	57,4
		75 a 84 anos		128	32,5
		≥ 85 anos		40	10,1
AUTOPERCEPÇÃO DA SAÚDE	Saúde comparada a outras pessoas de sua idade	Excelente/muito boa/boa		226	57,4
		Regular ou ruim		168	42,6
ATIVIDADES DE VIDA DIÁRIA (AVD)	AVD (instrumentais)	Deixou de fazer compras	Sim	85	21,6
			Não	309	78,4
		Deixou de controlar finanças	Sim	71	18,1
			Não	323	81,9
		Deixou de realizar pequenos trabalhos domésticos	Sim	80	20,3
			Não	314	79,7
	AVD (básicas)	Deixou de tomar banho sozinho	Sim	24	6,1
			Não	370	93,9
COGNIÇÃO	Ficando esquecido		Sim	103	26,1
			Não	291	73,9
	Esquecimento piorou nos últimos meses		Sim	68	17,3
			Não	326	82,7
	Esquecimento impede a realização de atividades do cotidiano		Sim	55	14,0
			Não	339	86,0
HUMOR	Desânimo, tristeza ou desesperança no último mês		Sim	109	27,7
			Não	285	72,3
	Perda de interesse ou prazer, no último mês, em atividades antes prazerosas		Sim	81	20,6
			Não	313	79,4
MOBILIDADE	Alcance, preensão e pinça	Incapaz de elevar os braços acima do ombro	Sim	35	8,9
			Não	359	91,1
		Incapaz de manusear ou segurar pequenos objetos	Sim	31	7,8
			Não	363	92,2
	Capacidade aeróbica e/ou muscular	Perda de peso não intencional, IMC < 22 kg/m^2^, circunferência da panturrilha < 31 cm ou velocidade da marcha (4m) > 5 segundos	Sim	49	12,4
			Não	345	87,6
	Marcha	Dificuldade para caminhar capaz de impedir atividades cotidianas	Sim	109	27,7
			Não	285	72,3
		Teve duas ou mais quedas no último ano	Sim	110	27,9
			Não	284	72,1
	Continência esfincteriana	Perde urina ou fezes, sem querer	Sim	117	29,7
			Não	277	70,3
COMUNICAÇÃO	Visão	Comprometimento da visão capaz de impedir a realização de atividades	Sim	80	20,3
			Não	314	79,7
	Audição	Comprometimento da audição capaz de impedir a realização de atividades	Sim	79	20,1
			Não	315	79,9
COMORBIDADES MÚLTIPLAS	Polipatologia	≥ 5 doenças crônicas ≥ 5 medicamentos diários Internação nos últimos 6 meses	Sim	83	21,1
	Polifarmácia				

Na EFS, 190 idosos (48,2%) foram identificados como sem fragilidade, 93 (23,6%) como aparentemente vulneráveis, 74 (18,8%) apresentavam fragilidade leve, 32 (8,1%) fragilidade moderada, e 5 idosos (1,3%) fragilidade severa. No IVCF-20, 207 idosos (52,5%) foram classificados como robustos, ou com baixo risco de fragilidade, 110 (28,0%) apresentaram risco moderado de vulnerabilidade clínico-funcional, e 77 (19,5%) alto risco.

A estatística Kappa revelou índice de concordância de 0,599 entre os instrumentos (Tabela 4). O coeficiente de correlação de Pearson entre os valores da EFS e do IVCF-20 foi 0,755 (p < 0,001).

**Tabela 4:** Análise de concordância para a classificação de fragilidade, segundo *Edmonton Frail Scale* e Índice de Vulnerabilidade Clínico-Funcional-20 em idosos comunitários, 2017.

	*Edmonton Frail Scale* (EFS)				Total
	Sem fragilidade		Fragilidade		
IVCF-20	(n)	(%)	(n)	(%)	
Sem fragilidade	271	85,5	46	14,5	317
Fragilidade	12	15,6	65	84,4	77

Kappa = 0,599 (p < 0,001).

Após análise múltipla, as variáveis que se mantiveram estatisticamente associadas à fragilidade, em ambos os instrumentos, foram: idade igual ou superior a 80 anos, história de acidente vascular encefálico, polifarmácia, autopercepção negativa de saúde, registro de queda e internação nos últimos 12 meses. Escolaridade inferior a quatro anos, doença osteoarticular e perda de peso foram associadas à fragilidade apenas pela EFS, enquanto ter cuidador esteve associado a maior risco de fragilidade apenas no IVCF-20 (Tabela 5).

**Tabela 5:** Fatores associados à fragilidade em idosos comunitários, segundo *Edmonton Frail Scale* e Índice de Vulnerabilidade Clínico-Funcional-20 (análise múltipla), 2017.

Variáveis independentes		Fragilidade *Edmonton Frail Scale*			Fragilidade pelo IVCF-20		
		RP	IC95%	p	RP	IC95%	p
Faixa etária				0,001			< 0,001
	≥ 80 anos	1,643	1,239 – 2,178		3,327	2,204 – 5,021	
	Até 79 anos	1			1		
Escolaridade				0,002			
	Até 4 anos	2,171	1,314 – 3,589				
	> 4 anos	1					
Doença osteoarticular				0,016			
	Sim	1,410	1,065 – 1,865				
	Não	1					
Acidente vascular encefálico				< 0,001			< 0,001
	Sim	2,139	1,484 – 3,082		2,546	1,619 – 4,004	
	Não	1			1		
Polifarmácia				0,001			0,004
	Sim	1,610	1,217 – 2,130		1,657	1,174 – 2,337	
	Não	1			1		
Autopercepção de saúde				< 0,001			< 0,001
	Negativa	3,115	2,085 – 4,654		3,294	2,081 – 5,213	
	Positiva	1			1		
Perda de peso				0,006			
	Sim	1,542	1,132 – 2,102				
	Não	1					
Possui cuidador							0,020
	Sim				1,615	1,078 – 2,419	
	Não				1		
Queda nos últimos 12 meses				0,037			0,029
	Sim	1,363	1,019 – 1,824		1,503	1,043 – 2,166	
	Não	1			1		
Internação nos últimos 12 meses				< 0,001			0,005
	Sim	1,825	1,382 – 2,409		1,715	1,181 – 2,490	
	Não	1			1		

RP: razão de prevalência.

## DISCUSSÃO

A comparação entre EFS e IVCF-20 mostrou concordância moderada e forte correlação positiva. A prevalência de fragilidade em idosos comunitários foi maior na EFS. Fatores demográficos, sociais, econômicos e relacionados a morbidades, bem como a utilização de serviços de saúde, influenciaram a fragilidade em idosos comunitários, mas a diferença na identificação dessas variáveis pelos instrumentos foi pequena.

A semelhança e a relevância dos principais componentes justificam a concordância moderada entre os instrumentos. Ambas as escalas avaliam cognição, independência funcional, humor e condições de saúde (ou presença de morbidades). De forma particular, a EFS avalia ainda suporte social, medicação, nutrição, continência urinária e desempenho funcional, enquanto o IVCF-20 contempla idade, autopercepção da saúde, mobilidade e comunicação^12^^–^^14^.

Os resultados deste estudo diferem dos apresentados em revisão sistemática e metanálise de estudos conduzidos na América Latina e no Caribe^26^, em que a prevalência de fragilidade em idosos comunitários brasileiros, identificada pela EFS, foi de 35,8%, com IC95% 30,6–41,2^26^. Quanto ao IVCF-20, embora já tenha sido validado no Brasil, faltam estudos de base populacional que o tenham aplicado^14^.

O ponto de corte pode explicar a diferença das prevalências encontradas nos dois instrumentos. Nesse sentido, o ponto de corte estabelecido para o ICVF-20 define menos idosos em um processo de triagem para avaliação especializada, identificando idosos com maiores necessidades, o que é positivo para a relação custo-benefício, pois é grande o custo da avaliação geriátrica ampla. Na atenção primária, essa é uma oportunidade de otimizar recursos, considerando que nem sempre há serviços de atenção especializada disponíveis.

A diferença entre alguns componentes das duas escalas também pode justificar a discrepância entre as prevalências. Enquanto a EFS inclui o componente “suporte social”, o IVCF-20 engloba “idade” e “comunicação”. Além disso, componentes semelhantes são abordados de maneira diferente. A EFS utiliza o teste do desenho do relógio para avaliar a “cognição”, e o IVCF-20 aborda a memória por meio da evocação de palavras. O teste do desenho do relógio exige conhecimento de números, e o baixo índice de escolaridade entre os idosos brasileiros pode comprometer o resultado. Portanto, o baixo desempenho nesse teste, que aumenta a prevalência de fragilidade, pode estar relacionado a dificuldades não necessariamente ligadas a um déficit cognitivo^13^.

A avaliação do “estado de saúde” pela EFS inclui internações nos últimos 12 meses, enquanto o ICVF-20, no componente “comorbidades múltiplas”, considera internações nos últimos seis meses. Quanto à “independência funcional”, ou “incapacidade funcional”, há diferenças entre os instrumentos: preparar refeição/cozinhar, locomover-se de um lugar a outro, usar o telefone, lavar roupa e tomar remédios estão presentes na EFS; lavar louça e tomar banho estão presentes no IVCF-20.

A EFS aborda, no componente “uso de medicamento”, a condição de esquecer de tomar remédios, não considerada no IVCF-20. No componente “desempenho funcional” da EFS, o teste “levante e ande” cronometrado tem distância aproximada de três metros e tempo percorrido estratificado em “0 a 10 segundos”, “11 a 20 segundos” e “maior que 20 segundos”. O IVCF-20, por sua vez, avalia se o tempo gasto no teste de velocidade da marcha em quatro metros é ou não superior a cinco segundos.

O IVCF-20 ainda difere da EFS ao incluir o componente “mobilidade”, que avalia a capacidade de elevar os braços acima do ombro, manusear ou segurar pequenos objetos, Índice de Massa Corporal, circunferência da panturrilha, dificuldade para caminhar capaz de impedir atividades cotidianas, quedas no último ano e incontinência fecal. O instrumento aborda também a polipatologia no componente “comorbidades múltiplas”.

Percebe-se, portanto, que a diversidade de características dos instrumento influencia a prevalência da fragilidade em idosos. Um estudo de revisão sistemática^27^ concluiu que componentes de fragilidade e indicadores correspondentes variam consideravelmente a depender do método utilizado pelo instrumento. Constata-se ainda que falta consenso sobre quais elementos devem ser considerados para predizer a fragilidade e, por conseguinte, aumentar a precisão no diagnóstico dessa condição^27^.

Os resultados do presente estudo mostram associação entre idade avançada e fragilidade, independentemente do instrumento. Já a associação com baixa escolaridade foi identificada apenas pela EFS. Outros estudos que compararam instrumentos^15^^,^^18^ também perceberam essa associação da fragilidade com aumento da idade e menor nível de escolaridade. Na Holanda, investigação longitudinal identificou, além da relação com baixa escolaridade, a associação entre baixa renda e fragilidade^28^.

O histórico de acidente vascular encefálico e quedas – fatores associados à fragilidade em ambos os instrumentos –, assim como a doença osteoarticular identificada pela EFS, está em consonância com outros estudos^4^^,^^6^^,^^7^^,^^15^. A doença osteoarticular e as sequelas do acidente vascular encefálico geram limitação funcional, comprometendo a realização de atividades básicas, instrumentais e avançadas antes desempenhadas sem restrições, e o risco de queda aumenta.

Os dois instrumentos também encontraram associação entre polifarmácia e fragilidade, resultado destacado no consenso sobre essa condição^3^ e também encontrado por outros autores^15^^,^^28^^,^^29^. Estudo francês, por exemplo, verificou efeitos independentes e combinados da polifarmácia e da fragilidade no risco de mortalidade em idosos^28^. Essa vulnerabilidade pode ser explicada pelas peculiaridades farmacocinéticas e farmacodinâmicas dos medicamentos no organismo em processo de envelhecimento, bem como pelos potenciais efeitos adversos da interação medicamentosa.

Os dois instrumentos evidenciaram ainda associação da fragilidade com autopercepção negativa de saúde, indicador que incorpora tanto componentes físicos, cognitivos e emocionais como aspectos relacionados ao bem-estar e à satisfação com a própria vida^22^^,^^23^^,^^30^. Trata-se de medida, portanto, capaz de predizer mortalidade, declínio da capacidade funcional e fragilidade em idosos.

A EFS encontrou também associação entre fragilidade e relato de perda de peso. O estado nutricional comprometido é um sinal importante da fragilidade em idosos, e a intervenção nutricional é uma modalidade não farmacológica capaz de corrigir a deficiência de macro e micronutrientes e, consequentemente, evitar a perda de peso que pode levar à síndrome da fragilidade^7^.

A associação entre a fragilidade e a presença do cuidador foi identificada somente pelo IVCF-20. É provável que se trate de uma situação de causalidade reversa, isto é: o idoso frágil necessita da presença de um cuidador para auxiliá-lo nas atividades demandadas diariamente^7^^,^^9^^,^^19^. Assim, a demanda ou a presença de um cuidador seriam marcadores de fragilidade já existente.

A internação hospitalar se mostrou associada à fragilidade nos dois instrumentos, resultado também destacado em metanálise^6^. Embora a presença de doenças crônicas não seja necessariamente acompanhada de fragilidade, episódios agudos de determinadas enfermidades ou exacerbação de condições crônicas podem aumentar o risco de eventos adversos à saúde^7^, resultando em fragilidade no idoso e, consequentemente, em desfecho clínico desfavorável, como hospitalização^2^^,^^6^. Ademais, a internação, por qualquer causa, gera importantes mudanças no cotidiano do idoso.

Comparar instrumentos capazes de detectar a fragilidade em idosos comunitários pode contribuir para a busca de uma ferramenta que possa ser aplicada sobretudo no primeiro nível de atenção à saúde e em locais com poucos profissionais especializados em geriatria. As escalas utilizadas mostraram-se similares na identificação de fatores associados ou marcadores de fragilidade, apesar de suas peculiaridades. Ambas podem ser úteis às equipes de saúde ao destacar os componentes que mais interferem na fragilização e identificar idosos que demandam assistência especializada. Em um contexto de poucos recursos, o IVCF-20 parece ser mais útil, por identificar um menor número de pacientes a serem encaminhados para avaliação geriátrica ampla.

O presente estudo apresenta algumas limitações. A principal delas é a não realização de uma Avaliação Geriátrica Abrangente, que permitiria outras análises simultâneas dos dois instrumentos. Todavia, esse procedimento já foi realizado previamente, na validação dos dois instrumentos, de forma separada. Como este é um estudo transversal, não é possível estabelecer relações de causalidade. Deve-se ponderar ainda que alguns componentes de ambos os instrumentos são autorrelatados e dependem da memória do idoso entrevistado ou de seu cuidador. Contudo, cabe ressaltar que o trabalho teve amostra aleatória representativa de idosos comunitários cuidadosamente avaliados por meio de instrumentos já validados e confiáveis.

## CONCLUSÕES

Embora os instrumentos EFS e IVCF-20 tenham apresentado concordância moderada e forte correlação positiva, bem como características similares para identificar associações, a prevalência de fragilidade apontada se mostrou discrepante. Esse resultado destaca a necessidade de padronizar o instrumento de aferição da fragilidade em idosos comunitários.
